# Supplementary material for: Effect of perioperative lidocaine infusion on the subjective quality of recovery after surgery: Protocol for an updated systematic review and meta-analysis
Source: PLoS One. 2025 May 13;20(5):e0323342. doi: 10.1371/journal.pone.0323342 (PMC12074320; doi:10.1371/journal.pone.0323342)
Supplement: S2 File — (DOCX) [file pone.0323342.s002.docx]

**S2 File. Search strategies for included databases**

**Pubmed search strategy**

#1 lidocaine [MeSH Terms]

#2 ((((((((((((((((Lignocaine[Title/Abstract]) or (2-(Diethylamino)-N-(2,6-Dimethylphenyl)Acetamide[Title/Abstract])) or (2-2EtN-2MePhAcN[Title/Abstract])) or (Lidocaine Hydrocarbonate[Title/Abstract])) or (Xyloneural[Title/Abstract])) or (Dalcaine[Title/Abstract])) or (Lidocaine Hydrochloride[Title/Abstract])) or (Lidocaine Monohydrochloride[Title/Abstract])) or (Xylocaine[Title/Abstract])) or (Lidocaine Sulfate (1:1[Title/Abstract]))) or (Lidocaine Monoacetate[Title/Abstract])) or (Lidocaine Carbonate (2:1[Title/Abstract]))) or (Lidocaine Carbonate[Title/Abstract])) or (Lidocaine Monohydrochloride, Monohydrate[Title/Abstract])) or (Xylocitin[Title/Abstract])) or (Xylesthesin[Title/Abstract])) or (Octocaine[Title/Abstract])

#3 #1 or #2

#4 "Surgical Procedures, Operative"[Mesh]

#5 ((((((((((((((Surgical Procedures[Title/Abstract]) or (Procedures, Surgical[Title/Abstract])) or (Procedure, Surgical[Title/Abstract])) or (Surgical Procedure[Title/Abstract])) or (Operative Procedures[Title/Abstract])) or (Operative Procedure[Title/Abstract])) or (Procedure, Operative[Title/Abstract])) or (Procedures, Operative[Title/Abstract])) or (Operative Surgical Procedure[Title/Abstract])) or (Operative Surgical Procedures[Title/Abstract])) or (Procedure, Operative Surgical[Title/Abstract])) or (Procedures, Operative Surgical[Title/Abstract])) or (Surgical Procedure, Operative[Title/Abstract])) or (Surgery, Ghost[Title/Abstract])) or (Ghost Surgery[Title/Abstract])

#6 #4 or #5

#7 (((((quality of recovery score[Title/Abstract]) or (QoR-40[Title/Abstract])) or (Quality of Recovery-40[Title/Abstract])) or (Quality of Recovery-15[Title/Abstract])) or (QoR-15[Title/Abstract])) or (Quality of Recovery scale[Title/Abstract])

#8 randomized controlled trial[Publication Type] or randomized[Title/Abstract] or placebo[Title/Abstract]

#9 #3 and #6 and #7 and #8

**Embase search strategy**

#1 'lidocaine'/exp

#2 'lignocaine':ab,ti or '2-(diethylamino)-n-(2,6-dimethylphenyl)acetamide':ab,ti or '2-2etn-2mephacn':ab,ti or 'lidocaine hydrocarbonate':ab,ti or 'xyloneural':ab,ti or 'dalcaine':ab,ti or 'lidocaine hydrochloride':ab,ti or 'lidocaine monohydrochloride':ab,ti or 'xylocaine':ab,ti or 'lidocaine sulfate (1:1)':ab,ti or 'lidocaine monoacetate':ab,ti or 'lidocaine carbonate (2:1)':ab,ti or 'lidocaine carbonate':ab,ti or 'lidocaine monohydrochloride, monohydrate':ab,ti or 'xylocitin':ab,ti or 'xylesthesin':ab,ti or 'octocaine':ab,ti

#3 #1 or #2

#4 'surgery'/exp

#5 'surgical procedures':ab,ti or 'procedures, surgical':ab,ti or 'procedure, surgical':ab,ti or 'surgical procedure':ab,ti or 'operative procedures':ab,ti or 'operative procedure':ab,ti or 'procedure, operative':ab,ti or 'procedures, operative':ab,ti or 'operative surgical procedure':ab,ti or 'operative surgical procedures':ab,ti or 'procedure, operative surgical':ab,ti or 'procedures, operative surgical':ab,ti or 'surgical procedure, operative':ab,ti or 'surgery, ghost':ab,ti or 'ghost surgery':ab,ti

#6 #4 or #5

#7 'quality of recovery score':ab,ti or 'qor-40':ab,ti or 'quality of recovery-40':ab,ti or 'quality of recovery-15':ab,ti or 'qor-15':ab,ti or 'quality of recovery scale':ab,ti

#8 'randomized controlled trial':ab,ti or 'randomized':ab,ti or 'placebo':ab,ti

#9 #3 AND #6 AND #7 AND #8

**Cochrane Library search strategy**

#1 MeSH descriptor: [Lidocaine] explode all trees

#2 (Lignocaine):ti,ab,kw or (Lidocaine Hydrocarbonate):ti,ab,kw or (Xyloneural):ti,ab,kw or (Dalcaine):ti,ab,kw or (Lidocaine Hydrochloride):ti,ab,kw or (Lidocaine Monohydrochloride):ti,ab,kw or (Xylocaine):ti,ab,kw or (Lidocaine Monoacetate):ti,ab,kw or (Lidocaine Carbonate):ti,ab,kw or (Lidocaine Monohydrochloride, Monohydrate):ti,ab,kw or (Xylocitin):ti,ab,kw or (Xylesthesin):ti,ab,kw or (Octocaine):ti,ab,kw

#3 #1 or #2

#4 MeSH descriptor: [Surgical Procedures, Operative] explode all trees

#5 (Surgical Procedures):ti,ab,kw or (Procedures, Surgical):ti,ab,kw or (Procedure, Surgical):ti,ab,kw or (Surgical Procedure):ti,ab,kw or (Operative Procedures):ti,ab,kw or (Operative Procedure):ti,ab,kw or (Procedure, Operative):ti,ab,kw or (Procedures, Operative):ti,ab,kw or (Operative Surgical Procedure):ti,ab,kw or (Operative Surgical Procedures):ti,ab,kw or (Procedure, Operative Surgical):ti,ab,kw or (Procedures, Operative Surgical):ti,ab,kw or (Surgical Procedure, Operative):ti,ab,kw or (Surgery, Ghost):ti,ab,kw or (Ghost Surgery):ti,ab,kw

#6 #4 or #5

#7 (quality of recovery score):ti,ab,kw or (QoR-40):ti,ab,kw or (Quality of Recovery-40):ti,ab,kw or (Quality of Recovery-15):ti,ab,kw or (QoR-15):ti,ab,kw or (Quality of Recovery scale):ti,ab,kw

#8 'randomized controlled trial':ti,ab,kw or 'randomised':ti,ab,kw or 'placebo':ti,ab,kw or 'randomly':ti,ab,kw

#9 #3 and #6 and #7 and #8
