# Supplementary material for: Effect of perioperative lidocaine infusion on the subjective quality of recovery after surgery: Protocol for an updated systematic review and meta-analysis
Source: PLoS One. 2025 May 13;20(5):e0323342. doi: 10.1371/journal.pone.0323342 (PMC12074320; doi:10.1371/journal.pone.0323342)
Supplement: Attachment — Submitted filename: Response to Reviewers.docx [file pone.0323342.s004.docx]

**Dear reviewer**

We have special gratitude to you, for devoting your valuable time and energy to review our work entitled “Effect of perioperative lidocaine infusion on the subjective quality of recovery after surgery: protocol for an updated systematic review and meta-analysis” for giving constructive comments and valuable guidance. In line with this, the authors had exhaustively demonstrated and addressed questions and comments raised by reviewers using point-by-point responses as stated below.

**Journal requirements:**

1. Please ensure that your manuscript meets PLOS ONE's style requirements, including those for file naming.

Response: We have carefully checked throughout the manuscript.

2. Your ethics statement should only appear in the Methods section of your manuscript. If your ethics statement is written in any section besides the Methods, please move it to the Methods section and delete it from any other section. Please ensure that your ethics statement is included in your manuscript, as the ethics statement entered into the online submission form will not be published alongside your manuscript.

Response: Thank you for your suggestion. We have included the ethics statement in the Methods section of our manuscript.

3. Please include a separate caption for each figure in your manuscript.

Response: Thank you for your suggestion. We have included a separate caption for each figure in our manuscript.

4. Please include captions for your Supporting Information files at the end of your manuscript, and update any in-text citations to match accordingly.

Response: Thank you for your suggestion. We have included captions for our Supporting Information files at the end of our manuscript and update any in-text citations to match accordingly.

**Review Comments to the Author**

**Reviewer #1**: This protocol has two notable limitations that should be addressed. First, restricting inclusion to English-language publications introduces potential selection bias and may miss valuable evidence from non-English speaking regions, warranting removal of this restriction. Second, the protocol should better contextualize itself in relation to existing meta-analyses, particularly Hung et al. (PMID: 34547603), by clearly differentiating its scope, addressing identified knowledge gaps, and incorporating subgroup analyses to explore previously noted heterogeneity - these additions would strengthen the protocol's potential contribution to the evidence base while avoiding redundant investigation.

Response: Thank you for your suggestion. First, we have removed the restriction of English-language publication. Second, we have added this discussion about the existing meta-analyses.

**Reviewer #2:**
1. Please supplement the detailed search strategies for each database.

Response: Thank you for your feedback. We have supplemented the detailed search strategies for each database.
2. In the sentence "In recent years, lidocaine, an amino-amide local anesthetic agent, have been extensively investigated for its anti-nociceptive...", the subject "lidocaine" is singular, so the verb should be "has" instead of "have".
 Response: Thank you for pointing this out. We have rewritten this sentence as recommended.
3. Inclusion and exclusion criteria: In the inclusion criteria, the classification of surgical types only differentiates between elective or emergency surgeries and general anesthesia, without considering the impact of differences in surgical sites and trauma severity on the results, which may increase the heterogeneity of the study. It is recommended to further refine the criteria related to surgical types. For example, studies could be included by classifying surgical categories such as abdominal, thoracic, and orthopedic surgeries. In the exclusion criteria, the description of "lidocaine being part of an opioid - free anesthetic technique" is rather ambiguous. Different studies may have different definitions of opioid - free anesthetic techniques. It is suggested to clearly define this criterion or explain how to judge it during the actual screening process.
 Response: Thank you for the insightful suggestion. First, we have revised the inclusion criteria as recommended. Second, we have modified the description of “lidocaine being part of an opioid - free anesthetic technique”, we appreciate your guidance.
4. Regarding the subgroup analysis, only the assessment of the intervention effect based on patient characteristics and primary outcomes was mentioned, but the specific grouping factors were not clearly defined. It is recommended to list possible subgroup analysis factors such as age, gender, type of surgery, etc., and explain the basis for grouping and the expected analysis objectives.
 Response: Thank you for the insightful suggestion. We have added a detailed description of the subgroup analysis for the systematic review and meta-analysis.
5. Discussion section: When pointing out the potential limitations of the study, issues such as heterogeneity, diversity of surgical types, and small sample size were only briefly mentioned, without in - depth exploration of how to minimize these impacts in subsequent studies. It is recommended to propose specific countermeasures for each limitation. For example, in the study design stage, how to optimize the inclusion criteria to reduce heterogeneity, and how to expand the sample size, etc. The description of the expected research results is rather general, only mentioning providing suggestions for clinical practice and research. It is recommended to clarify the specific forms of expected results, such as formulating the recommended dosage range for the perioperative use of lidocaine and a list of applicable surgical types.

Response: Thank you for the detailed guidance. We have revised the discussion section to propose specific countermeasures for each limitation. We have also addressed potential limitations and how the findings will be interpreted and disseminated.
