# Supplementary material for: Effect of perioperative lidocaine infusion on the subjective quality of recovery after surgery: Protocol for an updated systematic review and meta-analysis
Source: PLoS One. 2025 May 13;20(5):e0323342. doi: 10.1371/journal.pone.0323342 (PMC12074320; doi:10.1371/journal.pone.0323342)
Supplement: Attachment — Submitted filename: Response_to_Reviewers_auresp_2.docx [file pone.0323342.s005.docx]

**Dear Editors and Reviewers:**

Thank you very much for giving us the opportunity to revise our manuscript (PONE-D-24-41550R1). We appreciate the helpful feedback from you and the reviewers. After carefully reading the comments, we have revised the manuscript point-by-point. Herewith we resubmit a revised manuscript for your assessment. Important changes are highlighted, and detailed responses to each comment are included below. We believe that these revisions have substantially improved the manuscript, and we thank you and the reviewers for their thoughtful comments, which are really helpful for the improvement.

Journal Requirements:
Please review your reference list to ensure that it is complete and correct. If you have cited papers that have been retracted, please include the rationale for doing so in the manuscript text, or remove these references and replace them with relevant current references. Any changes to the reference list should be mentioned in the rebuttal letter that accompanies your revised manuscript. If you need to cite a retracted article, indicate the article’s retracted status in the References list and also include a citation and full reference for the retraction notice.

Response: Thank you! We have checked the reference list to ensure that it is complete and correct.

Reviewer #2: The article has been revised very well, and I highly appreciate the efforts made by the authors. However, there is still a minor issue that needs to be addressed.

Data Extraction and Analysis: The data extraction and bias risk assessment were carried out by two independent personnel, and the methodology is reasonable. In the data extraction form, an item of "specific infusion regimen of lidocaine (such as infusion rate and concentration)" can be added to enable a more comprehensive analysis of the impact of lidocaine use on the results. When conducting subgroup analysis, in addition to the factors mentioned in the article, the factor of different anesthesia methods can be considered for inclusion, because the anesthesia method may affect the efficacy of lidocaine.

Response: Thank you very much for your valuable comments. We have added the regimens of lidocaine infusion in the data extraction form and anesthesia methods in subgroup analysis.
